# Traumatic Multi-Finger Amputations After Trying to Rein in a Horse

**DOI:** 10.7759/cureus.5171

**Published:** 2019-07-18

**Authors:** Rohan Mangal, Tej G Stead, Latha Ganti, Travis Jasper, Bryan C Sleigh

**Affiliations:** 1 Emergency Medicine, John Hopkins University, Baltimore, USA; 2 Emergency Medicine, Brown University, Providence, USA; 3 Emergency Medicine, Envision Physician Services, Orlando, USA; 4 Emergency Medicine, Wellstar Atlanta Medical Center, East Point, USA; 5 Emergency Medicine, Mercer University School of Medicine, Macon, USA

**Keywords:** digit amputation

## Abstract

The authors present a case of traumatic, multiple partial-digit amputations caused by the patient entangling three of his fingers in the reins of a moving horse. The patient experienced amputation in fingers III-V on the dominant, right hand. The patient was stabilized and provided analgesia before referral to a surgical center for potential replantation. The emergency management of traumatic finger amputations is reviewed.

## Introduction

Traumatic amputations represent approximately 1% of all trauma-related injuries. Among the said cases, multiple digit amputations are far less common [1]. In the management of finger amputation, the foremost goal is to restore function in severed parts. Patients with partial-hand amputation experience reduced grip strength and functionality, exacerbated by when fingers III-V are removed [2].

The goals of treating a fingertip amputation are to (1) preserve functional length, (2) achieve durable coverage, (3) achieve near-normal sensibility, (4) prevent adjacent joint contracture, (5) achieve early functional recovery, and (6) provide adequate pain control [3-4].

Replantation can provide a tremendous quality-of-life difference in patients psychologically and functionally. The success of a digit replantation is often contingent on how well-preserved the extremity is. It is well-supported that digit replantation is optimistic within 24 hours of cold ischemia time and 12 hours of warm ischemia time [5]. However, freezing the amputated extremity in water is not recommended [6].

## Case presentation

A 25-year-old healthy male, who is right hand dominant, presented to our freestanding emergency department (ED) with right, third, fourth, and fifth finger trauma. The 3rd and 4th digits sustained partial amputations. The 5th digit sustained a complete fingertip amputation distal to the distal interphalangeal joint. The patient’s case was the result of helping his friend with a pet horse. The injury occurred from entangling fingers III-V in the reins of the horse before it began galloping away. The degree of bleeding was moderate and the patient reported moderate pain from the incident. There were neither exacerbating factors nor relieving factors. Aside from pain, there were no other related symptoms. Past medical and surgical history was negative; he had no drug allergies and denied any toxic habits.

**Figure 1:**
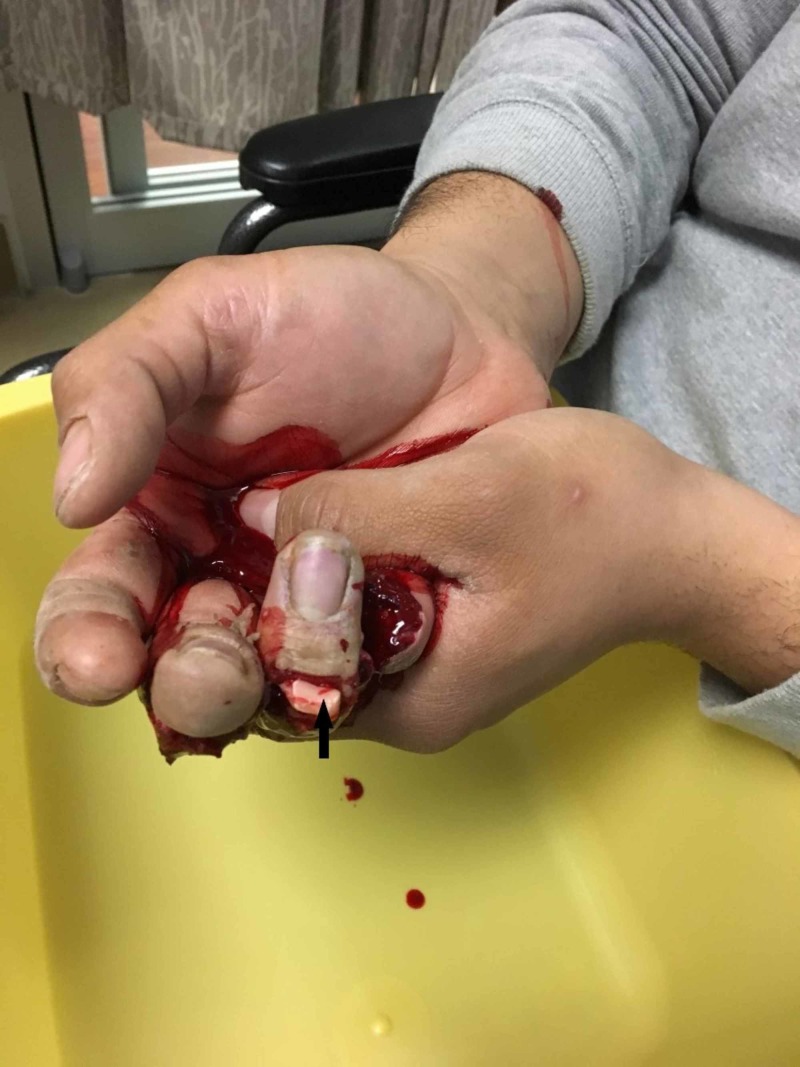
Aerial view of multiple digit amputation Arrow denotes severed tendon.

**Figure 2:**
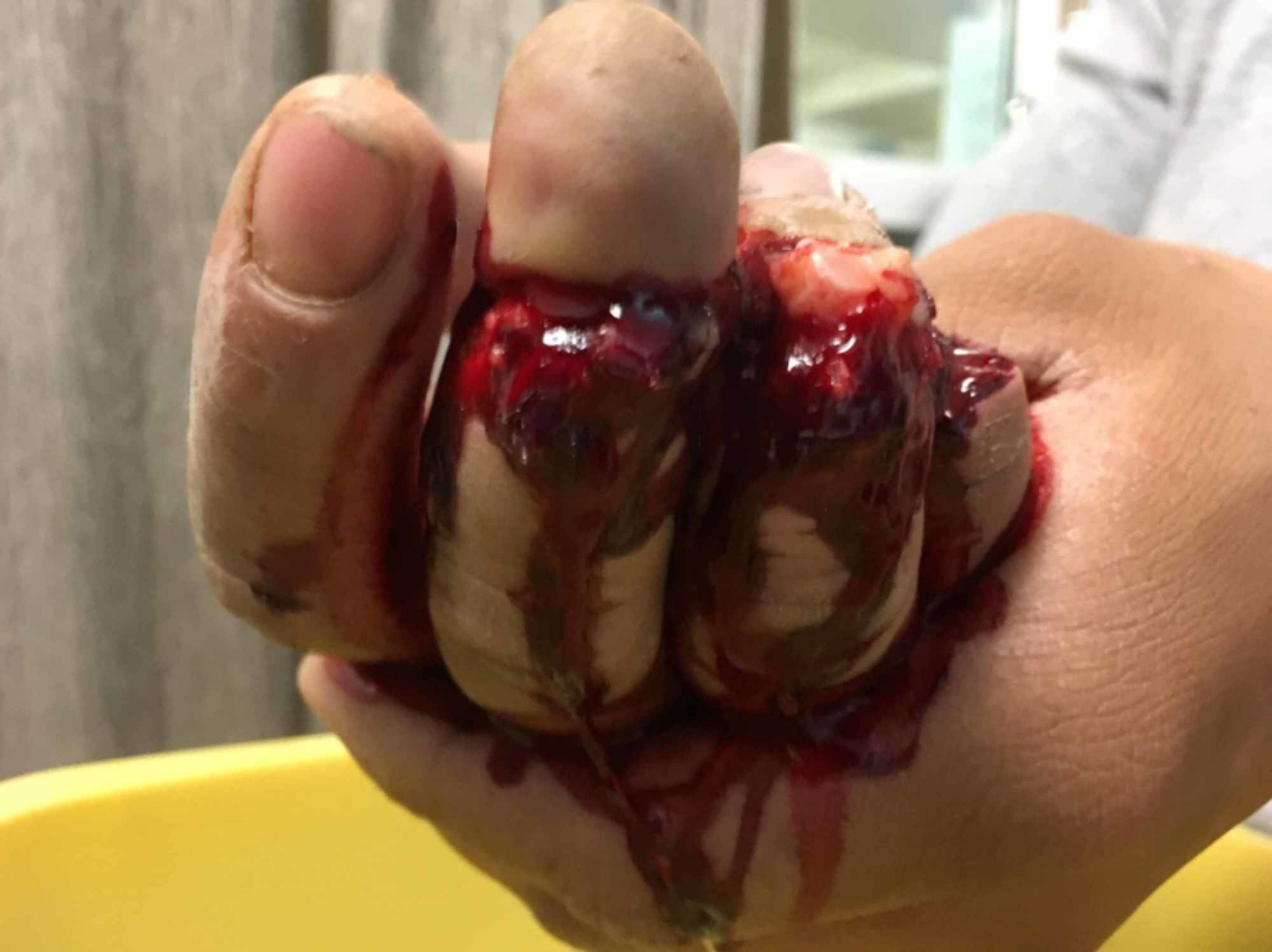
Rear view of multiple digit amputation

**Figure 3:**
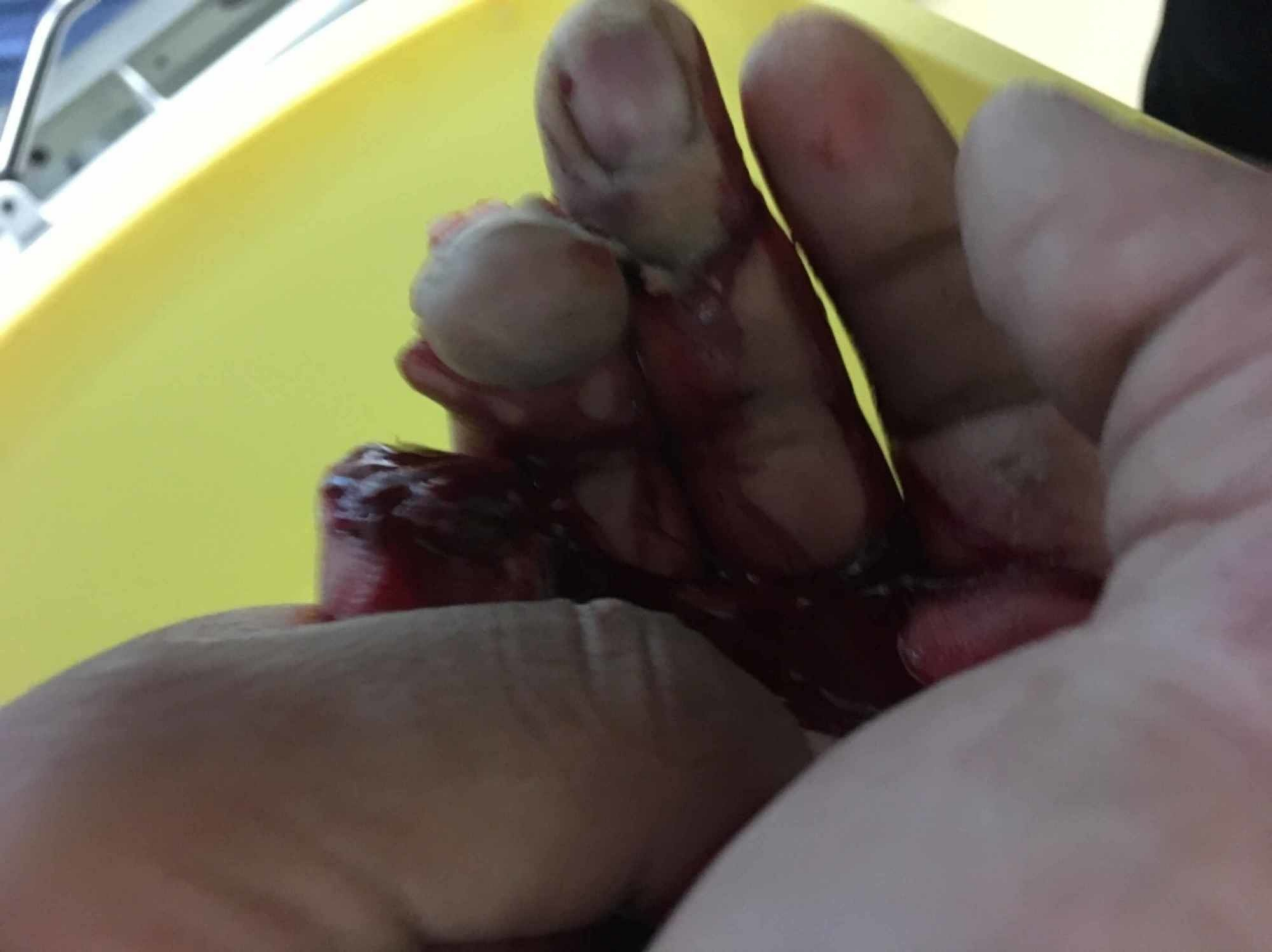
View of multiple digit amputation from palm of hand

The patient arrived with a makeshift dressing of his hand and holding the severed distal fingertip in his shirt pocket. The dressing was removed and the hand examined. He had an intact radial pulse, and the hand was overall warm and well perfused. He was unable to fully straighten his middle three fingers at the proximal interphalangeal joints. The exposed tendon was visible in digit IV (Figure 1). The tip of digit III was twisted 180° such that the nail was visible on the palmar rather than the dorsal aspect of the hand (Figures 2, 3).

The patient’s last tetanus was at age 15, and so he was given tetanus toxoid. For analgesia, he received 4mg of morphine intravenously (IV) and a digital block of fingers III-IV was performed using 1% lidocaine without epinephrine. One gram of cefazolin IV was given as antibiotic prophylaxis. The severed part was wrapped in saline-moistened gauze and placed in a clean re-sealable plastic bag.

A call was then made to the local trauma center, where he was accepted by the hand surgeon on call. On follow-up, we learned that the patient’s fourth fingertip was successfully replanted.

## Discussion

The emergency management of traumatic finger amputation consists of stabilizing the patient, preserving the severed part if available, and prompt referral to a hand specialist. The best medium for a severed finger is to wrap it in saline-moistened gauze [1,3]. Placing the part directly on ice is not recommended. The optimal analgesia for hand injuries is to provide a digital block. The traditional teaching is that epinephrine should not be used in fingers due to the fear that it could result in finger necrosis or gangrene. However, several comprehensive literature reviews have debunked this myth [7-8].

Patients who incur a multiple digit amputation may experience a significant decrease in hand functioning and grip strength. This injury poses great psychological and social burden and can cause a marked drop-off in quality-of-life (QOL) in both males and females [6].

Replantation is the process of reattaching severed body parts to restore function. Indications for digit replantation include severance of thumb or multiple fingers, as well as amputation at or proximal to the palm. Digit replantation is also considered with a finger amputation at any level in pediatric patients [9].

## Conclusions

When working with potentially dangerous animals, it is important to take proper safety precautions. Amputation of fingers III-V can profoundly lower patients’ quality-of-life and demand immediate attention. Replantation should be considered after assessing patient eligibility through indications to restore function in severed body parts. To ensure the success of such a surgical intervention, amputated parts should be preserved in cold saline to maximize ischemic tolerance times.
